# DefiPace^TM^ System, A New Device for Cardioversion of Atrial Fibrillation After Cardiac Surgery — Preliminary Results

**DOI:** 10.31083/j.rcm2304143

**Published:** 2022-04-12

**Authors:** Helmut Mair, Ferdinand Vogt, Johannes Göppl, Evgeny Goldin, Dow Rosenzweig, Paul Kofler, Guiseppe Santarpino, Peter Lamm

**Affiliations:** ^1^Department of Cardiac Surgery, Artemed Klinikum München Süd, 81379 Munich, Germany; ^2^Department of Cardiac Surgery, Paracelsus Medical University, 90471 Nuremberg, Germany; ^3^Department of Cardiology, Artemed Klinikum München Süd, 81379 Munich, Germany; ^4^Faculty of Medicine, Ludwig Maximilians University of Munich, 81379 Munich, Germany; ^5^Cardiac Surgery Unit, Department of Experimental and Clinical Medicine, University “Magna Graecia" of Catanzaro, 88100 Catanzaro, Italy

**Keywords:** atrial fibrillation, postoperative atrial fibrillation, POAF, temporary atrial pacing wires, postoperative cardioversion

## Abstract

**Objectives:**

Postoperative atrial fibrillation (POAF) is a frequent complication following cardiac surgery. This study examined the safety and efficacy of the new ${\mathrm{DefiPace}}^{\text{TM}}$ system consisting of two bi-atrial temporary pacing and cardioversion electrodes, a ventricular electrode and the ${\mathrm{DefiPace}}^{\text{TM}}$ device (combined external pacemaker and cardioverter) for low-energy atrial cardioversion.

**Methods:**

The temporary electrodes were placed on the left and right atrium during open heart surgery. Pacing thresholds and sensing were measured up to the 6th postoperative day. The satisfactory handling of the electrodes was measured with a visual analog scale (VAS) 1–10, with 10 being the best and 1 being the lowest. In case of POAF, R-wave synchronous low-energy shocks (0.5–10 J) were applied for cardioversion.

**Results:**

Temporary electrodes were implanted in 29 patients (age 65.6 ± 10.4 years; 21 males, 14 OPCAB, 15 on-pump cardiac operations). Left or right atrial pacing thresholds ranged from 1.9 ± 1.3 V/ms to 5.0 ± 3.3 V/ms and P-wave sensing from 0.9 ± 0.6 mV to 1.5 ± 0.7 mV. VAS for handling of electrodes: implantation 7.1 ± 0.8 and removal 8.4 ± 1.0. POAF was observed in four patients. Two patients had successful atrial cardioversion with 3.5 J and 4.5 J. One patient converted spontaneously, and one patient remained in PAOF. There were no device-related adverse events.

**Conclusions:**

The ${\mathrm{DefiPace}}^{\text{TM}}$ system can be used safely in patients undergoing cardiac surgery.

## 1. Introduction

Postoperative atrial fibrillation (POAF) is a frequent (20–70%) complication following cardiac surgery, often resulting in prolonged hospital stay and an increased risk of morbidity and mortality [1, 2]. POAF tends to occur one to three days after cardiac surgery, with a peak incidence on postoperative day two [3]. Standard-of-care is to treat POAF using high-dose anti-arrhythmic medication (beta-blockers, amiodarone, magnesium) and external high-energy atrial cardioversion, all of which have either side effects or are particularly time-consuming [1, 2]. Bi-atrial pacing, as a measure to prevent the onset of POAF, has been shown to be successful in many clinical studies and meta-analyses [4, 5, 6, 7, 8, 9, 10, 11, 12], and have been included in the European Guidelines for cardiac surgery patients, even in the absence of a commercially available bi-atrial pacemaker device. Similarly, low-energy internal cardioversion of POAF has also been used for some time [13, 14, 15, 16, 17, 18, 19, 20], and good results have already been achieved with this technique. However, the procedures were less practicable because they required complex fixation of the electrodes, and two separate devices were required for postoperative pacing and low-energy cardioversion. This and the lack of practicable product lines on the market are the likely reasons why both methods of bi-atrial pacing and internal cardioversion, both of which are more comfortable for the patient, did not prevail despite good results.

This study aimed to examine the safety and efficacy of low-energy atrial defibrillation using a new system named ${\mathrm{DefiPace}}^{\text{TM}}$ (Osypka AG, Rheinfelden, Germany) consisting of two temporary TMA® (Temporary Myocardial Atrial) electrodes (Osypka AG, Rheinfelden, Germany) for pacing and cardioversion and the hand-held ${\mathrm{DefiPace}}^{\text{TM}}$ external pacemaker and cardioverter device.

## 2. Material and Methods 

This patient cohort analysis is a retrospective observation of 29 patients after cardiac surgery (age 65.6 ± 10.4 years; 21 males). All patients were in sinus rhythm (SR) at the time of hospital admission. Three patients had a history of paroxysmal atrial fibrillation (AF). Patients with implantable electrical devices were not included. All investigations that were performed were within the scope of the intended use of the device.

The study was conducted in two phases. Phase one was initiated when the atrial electrodes had initial CE approval (since November 2016) for single or bi-atrial pacing only. In this phase one, the safety and efficacy of the electrodes were observed and thus served as an internal safety control. Electrodes were placed either on the right or left atrium or both. In the second phase starting in 2020 (after CE certification for additional atrial defibrillation in 2019), the bi-atrially implanted electrodes were also used for atrial cardioversion in case of POAF. The study protocol conforms to the ethical guidelines of the Declaration of Helsinki and the study complies with the local medical board’s ethical regulations (Bayerische Landesärztekammer No.: 2021-1067).

**Fig. 1.**
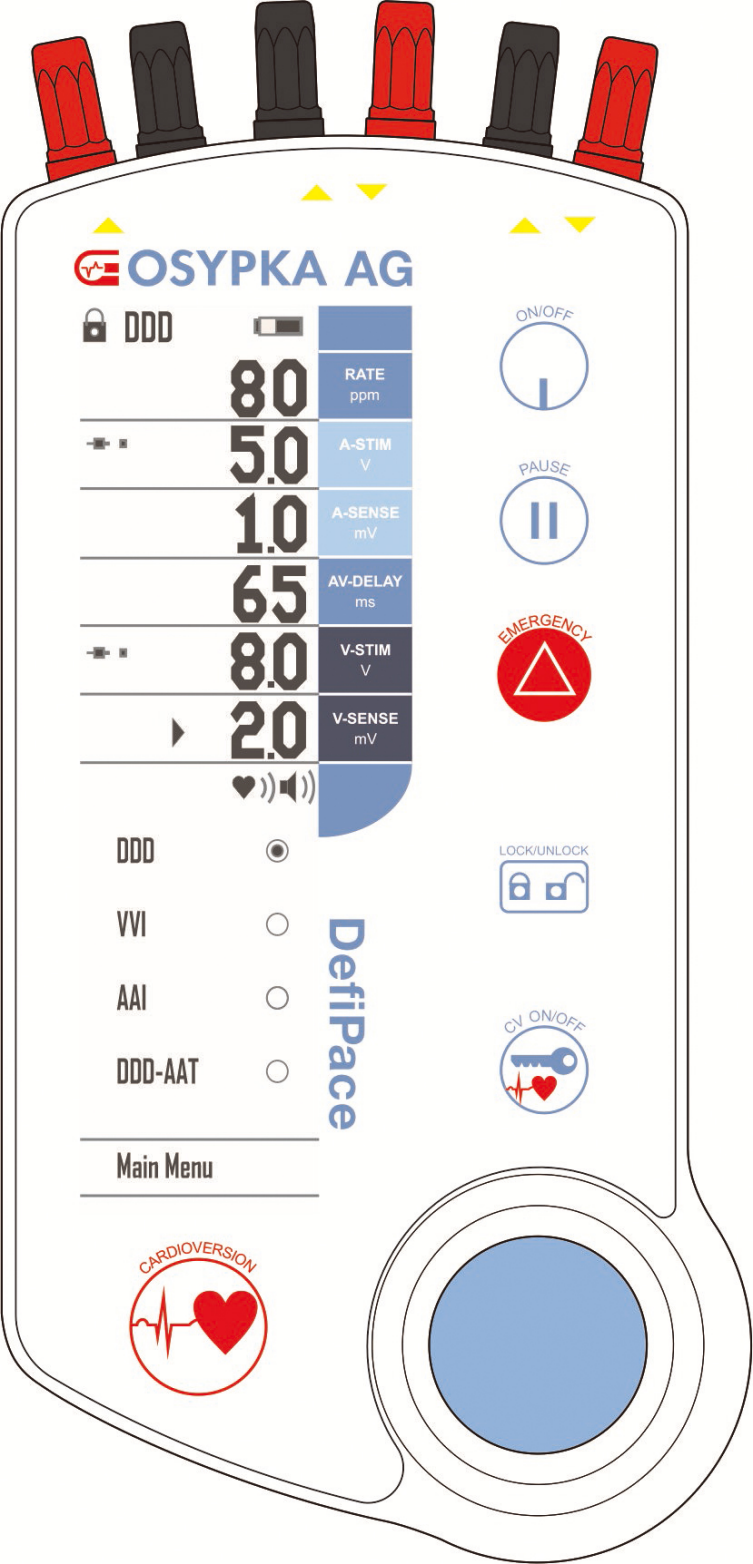
**Osypka ${\mathrm{DefiPace}}^{\text{𝐓𝐌}}$-Device (Osypka AG, Rheinfelden, Germany)**. An external three-chamber pacemaker for bi-atrial pacing and low-energy cardioversion (the two left plugs for left atrial, the two right plugs for right atrial and the middle two plugs for the ventricular electrodes).

The ${\mathrm{DefiPace}}^{\text{TM}}$ is a three-chamber pacemaker combined with a dual-chamber low-energy cardioverter for temporary atrial cardioversion and atrial synchronization (Fig. 1).

**Fig. 2.**
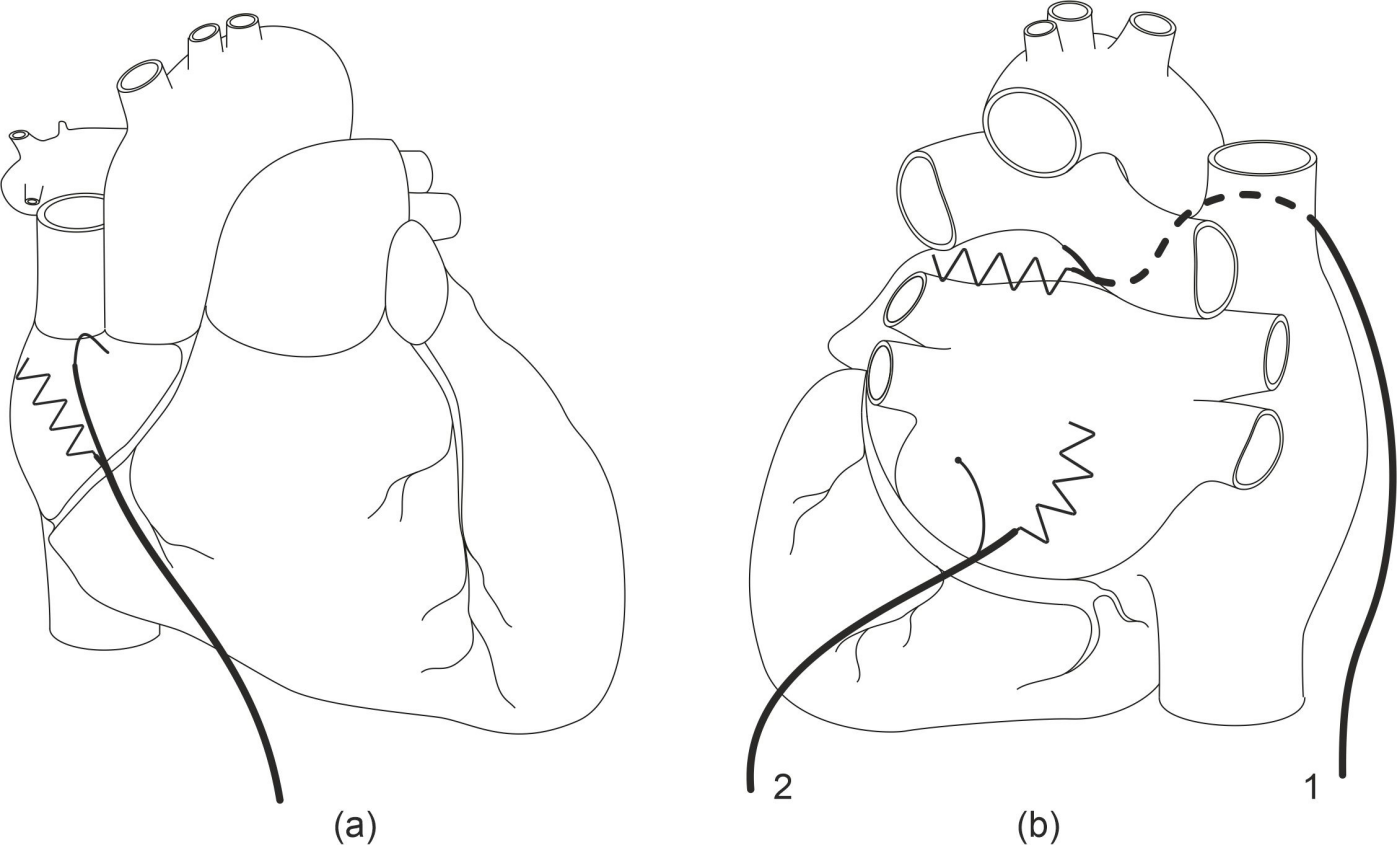
**Schematic drawing of a human heart [front (a) and back view (b)] showing the atrial temporary wire electrodes in place**. The distal 10 cm of the electrode (anode) formed as a zigzag were placed at the right (a) and left atria (b), respectively. On the right side (b), back view of the heart, the electrodes were either placed via transvers sinus (1) or fixed on the left atrium (2).

The ${\mathrm{DefiPace}}^{\text{TM}}$ system requires a temporary (right) ventricular bipolar pacing wire [TME (temporary myocardial electrode), Osypka AG, Rheinfelden, Germany] and two atrial TMA® wires, one on each atrium. All three wires are connected via corresponding extension cables to the external ${\mathrm{DefiPace}}^{\text{TM}}$, which received CE market approval in 2017. If only DDD-pacing is chosen, the atrium and ventricle electrodes can also be connected to a standard external pacemaker (e.g., Osypka Pace203H, Osypka AG, Rheinfelden, Germany). The stainless-steel bipolar pacing wires are insulated 60 cm electrodes. The defibrillation electrode, which is also the anode, has 10 cm of uncoated wire at its distal portion, formed in a zigzag-shape, to ensure a suitable surface for shock delivery (Fig. 2). The 5 mm long cathode (pacing/sensing electrode) is located more proximally, and the insulation of both electrical conductors is welded together.

There are different types of TMA® electrodes available. They vary only in the fixation mechanisms: the ends of the cathode or anode are available with or without a needle. The type of electrode is chosen by physician preference. The operating function is identical.

**Fig. 3.**
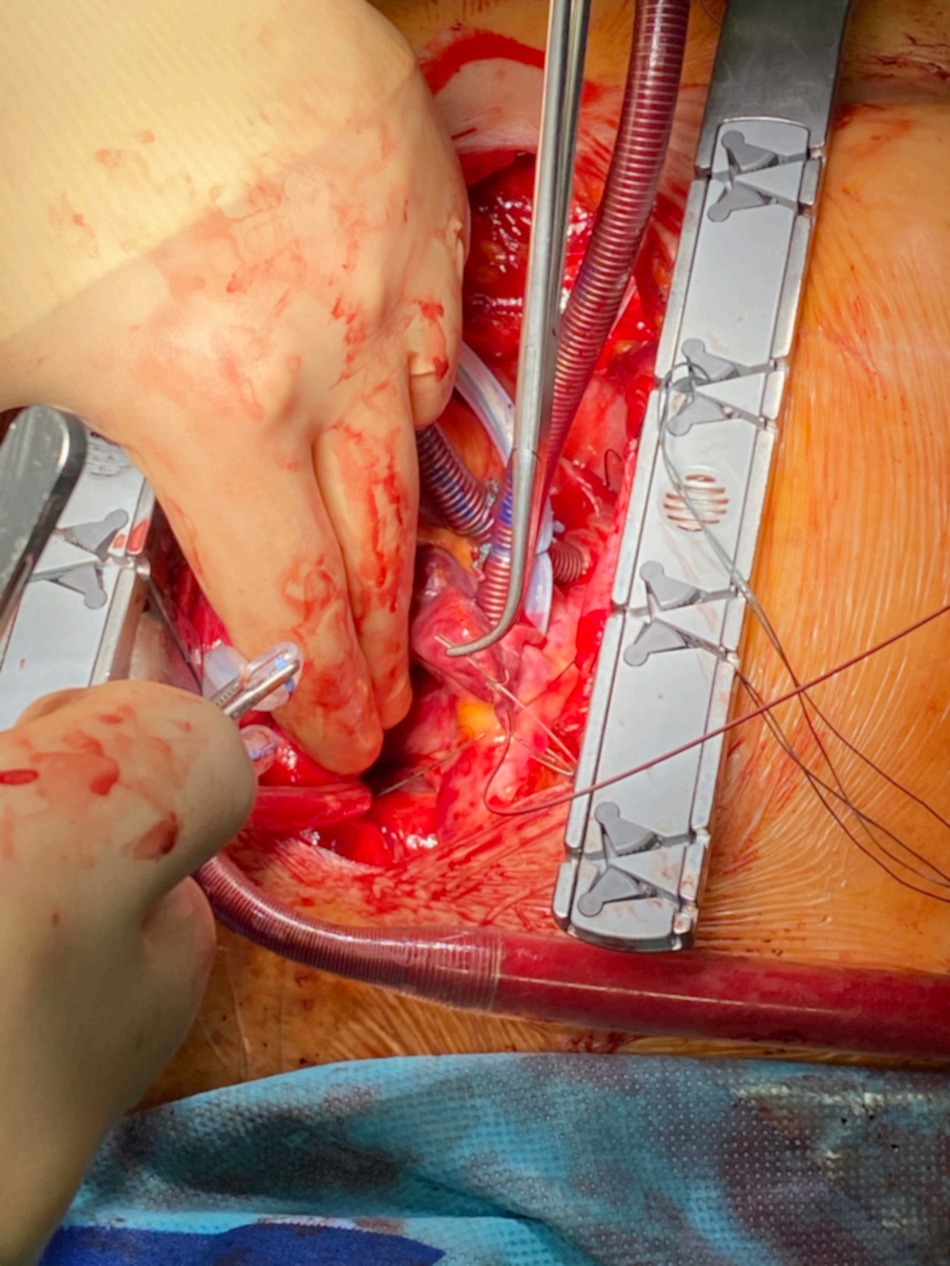
**Implantation of the atrial TMA® electrodes without needle**: placement of the TMA® wire transferred through the transvers sinus for placement at the left atrium without fixation.

**Fig. 4.**
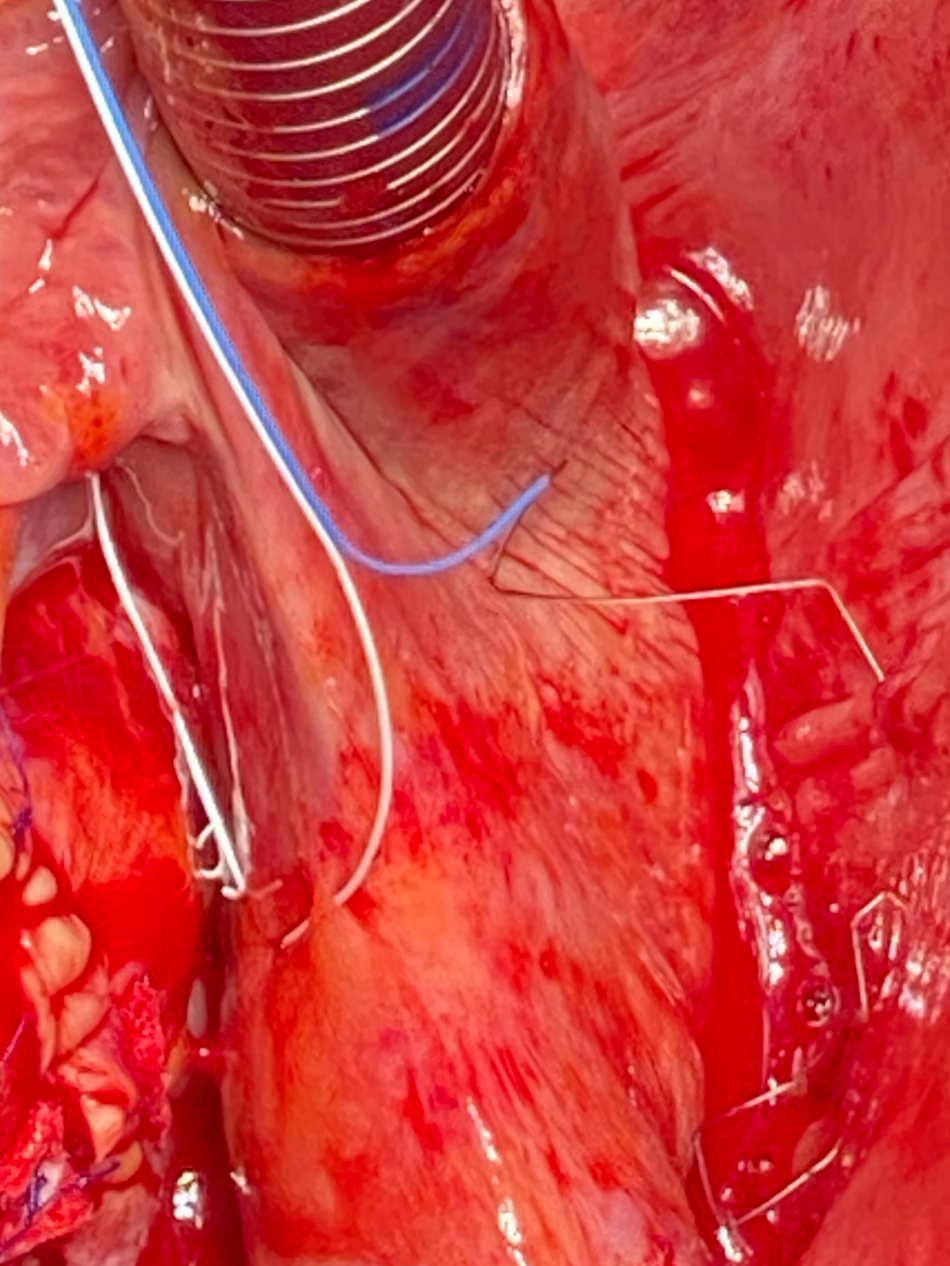
**Implantation of the atrial TMA® electrodes with needles**: placement of the right wire with anode stitched to the pericardium and the cathode on the superior vena cava.

The temporary left atrial electrodes were either transferred through the transverse sinus for placement at the left atrium without fixation (Figs. 2b, 3) or fixated on the epicardial or pericardial surface based on the preference of the individual surgeon. In case the electrodes were fixed, the anode (defibrillation zigzag) was either placed and fixed between the free wall of the left atrial appendage or fixed to the pericardium and the left upper pulmonary vein (Fig. 2b). The cathode was placed one to two cm distal from the anode. The anode of the TMA® wire to the right atrium was placed and fixated to the free right atrial wall between the superior and inferior vena cava (Fig. 4); and the cathode was placed at the sinus node one to two cm distal to the anode. The electrodes were placed and stitched with meticulous care at the atria, and guided carefully in the pericardium to ensure smooth extraction. Special attention was given to bypass grafts to prevent damage during extraction. The proximal ends of the electrodes were lead through the skin of the patient’s chest and secured with a suture. The three connection cables (two from TMA® wires and one from the ventricular wire) are then plugged into the external device, Osypka ${\mathrm{DefiPace}}^{\text{TM}}$. It is possible to set the pacing program for bi-atrial pacing, and additionally, in case of POAF, to apply an internal low energy cardioversion shock impulse to both atria (up to max. 10 J), triggered with the ventricular lead, to ensure R-wave synchronization of the atrial cardioversion. Ventricular and atrial pacing is possible with a maximum of 18 V/0.5 ms.

Postoperatively, the pacing threshold and sensing (P- and R-wave amplitudes) were measured in a standard manner up to the 6th postoperative day with the ${\mathrm{DefiPace}}^{\text{TM}}$ or the Osypka Pace203H. When POAF occurred, a bi-atrial R-wave synchronous shock was applied with the ${\mathrm{DefiPace}}^{\text{TM}}$, starting with 3.5 J. In case of ongoing POAF, the energy was increased stepwise by increments of one Joule. Low-dose propofol was given before internal defibrillation. Between postoperative day 5 and hospital discharge, all electrodes were removed by transcutaneous extraction. Immediately after extraction the wires were inspected for integrity. The surgeons rated the extraction of the temporary electrodes using a visual analog scale (VAS) with 1 (very difficult, adverse event or patients’ discomfort) to 10 (very easy without any problems). Data were presented as mean ± SD.

## 3. Results 

Twenty-nine patients (mean age: 64.6 ± 10.8 years; male/female = 21/8) were operated on in a standardized manner using a median sternotomy or partial upper mini-sternotomy. Fourteen patients underwent off-pump coronary artery bypass (OPCAB) revascularization. Fifteen patients received on-pump cardiac operations using cold crystalloid cardioplegia with six patients receiving bypasses, four patients having valve surgery, and five patients having combined procedures.

After training, the implantation of the TMA® electrodes took 4.2 ± 2.7 min with 16 TMA® electrodes placed in the first period (eight via the transverse sinus and eight right atrial) and 13 patients were observed in the second period (bi-atrial placement of TMA® electrodes). All right atrial electrodes were placed as described in the methods section. In total, a left atrial electrode was placed in 14 patients via the transverse sinus. In 7 cases the anode (zigzag) was placed and fixed between the free wall of the left atrial appendage and the pericardium. The surgeons rated the implantation with VAS 7.1 ± 0.8.

The sensing and the threshold of the electrodes are shown in Table 1. In all patients, the atrial pacing and sensing values for left and right atrial threshold and sensing were acceptable, and pacing was possible except when POAF occurred.

**Table 1.** **Sensing and the threshold of the electrodes up to 6th postoperative day**.

	sensing threshold			pacing threshold		
	TMA right atrium (mV)	TMA left atrium (mV)	Ventricular electrode (mV)	TMA right atrium (V/0.5 ms)	TMA left atrium (V/0.5 ms)	Ventricular electrode (V/0.5 ms)
OD	1.0 ± 0.6	1.5 ± 0.7	4.7 ± 3.0	1.9 ± 1.3	3.9 ± 3.1	1.9 ± 1.4
1. pod	0.9 ± 0.6	1.3 ± 0.6	4.4 ± 3.2	2.1 ± 1.5	3.9 ± 1.9	2.1 ± 1.6
2. pod	0.9 ± 0.6	1.0 ± 0.4	3.3 ± 2.7	2.5 ± 1.8	4.4 ± 2.2	2.6 ± 2.3
3. pod	1.0 ± 0.5	1.1 ± 0.4	2.7 ± 1.5	2.7 ± 1.8	4.3 ± 2.5	3.1 ± 2.0
4. pod	1.0 ± 0.7	1.2 ± 0.5	2.7 ± 1.3	2.5 ± 2.0	5.0 ± 2.5	3.2 ± 1.6
5. pod	1.2 ± 0.7	1.1 ± 0.6	3.0 ± 2.3	2.8 ± 2.3	4.8 ± 3.2	3.4 ± 1.9
6. pod	1.3 ± 0.7	1.1 ± 0.7	3.3 ± 2.2	2.9 ± 2.2	5.0 ± 3.3	3.4 ± 1.7

Legend: data are expressed as mean ± standard deviation. TMA, temporary myocardial atrial (electrodes); OD, day of operation; pod, postoperative day.

In 4 patients, POAF was observed between 2–4 postoperative days. In two patients, POAF was converted to sinus rhythm using the ${\mathrm{DefiPace}}^{\text{TM}}$. In one patient (male, 63 years old, OPCAB, no history of AF) 3.5 J (2 × 3.5 J within 5 minutes) and in the other patient 4.5 J (male, 78 years old, CABG, no history of AF) were applied. In another patient cardioversion was impossible because the ventricular lead did not trigger due to low sensing of the ventricular lead. To trigger the internal atrial defibrillation, the ${\mathrm{DefiPace}}^{\text{TM}}$ must detect the R-wave with >1 mV. This patient was then converted with antiarrhythmic medication (amiodarone and ß-blocker). In one patient, the POAF spontaneously converted to sinus rhythm shortly before shock delivery. Therefore, the ${\mathrm{DefiPace}}^{\text{TM}}$ shock application procedure was interrupted and thus no shock was applied.

The wires were removed without complication after 6.2 ± 1.0 days postoperatively by external pulling on the electrodes in all patients. The extraction of the wires was easy and complete in all cases. Surgeons rated the extraction of the temporary TMA® electrodes with VAS 8.4 ± 1.0. Only one extraction was rated three, which needed a second uneventful attempt by an experienced surgeon and analgesia for the patient, most likely due to accidental fixation with the sternal wires. No device-related complications occurred during shock application or with lead extraction. There were no device-related adverse events during the in-hospital stay and within 6 weeks of follow-up. No wound infections related to the TMA® electrodes occurred. Two patients received antibiotic therapy postoperatively due to elevated inflammatory values without microbial or clinical evidence for a bacterial infection. One patient was treated with topical dressing changes due to a poorly healing wound involving the lateral drainage port and another at the upper pole of the thoracic incision. No patient died or needed reoperation due to complications involving the TMA® wires. One patient had a surgical revision on the 3rd postoperative day due to bleeding from an unclipped side branch of a bypass graft unrelated to the TMA® wires. No patients received bi-atrial pacing postoperatively, since the focus of this study was on the safety of the implantation of the electrodes, their sensing and pacing thresholds, and their ability to cardiovert patients with POAF.

## 4. Discussion

This study proved the safety and pacing efficacy of left and right atrial TMA® electrodes during cardiac surgery. Low-energy atrial cardioversion for postoperative atrial fibrillation using epicardial pacing wires was first described in 1998 by Liebold *et al*. [14] in a study with 238 patients. Additional studies by the same team were performed within the next year [16]. In these studies the wires (so-called TADpole wires) were similar to bipolar Osypka TME pacing wires, but featured a 10 cm long portion of uninsulated wire distally to the heart needle. The uninsulated wire portions for pacing and cardioversion had to be sutured onto the left and right atria with several stitches [13, 14, 16]. In contrast to the ${\mathrm{DefiPace}}^{\text{TM}}$ system used in our study, in these earlier studies, two devices had to be used, an external pacemaker for AV pacing, and for atrial cardioversion, the pacemaker had to be removed and an external defibrillator had to be connected.

Another multicenter European trial conducted by Kleine *et al*. [15] with a total of 296 patients, also using TADpole wires, also showed the suitability of using epicardial pacing wires for conversion of atrial fibrillation. Sixty-five patients had a total of 83 episodes of AF treated by TADpole wires with a conversion rate of 88.5%, using an energy of 6.0 ± 2.0 J, without clinical complications. The shocks were well tolerated with slight sedation. An additional prospective study with 145 patients using a control group with conventional pacing wires (no left atrial wires) was subsequently performed and demonstrated that no TADpole patient had longer than 24 hours of POAF, which led to a significantly reduced mean duration of AF in these patients. In the control group, conventional treatment added more than two days to the period of POAF, which was highly significant. There was no increase in risks, e.g., bleeding [17]. A similar significant result in the reduction of AF burden was achieved in another study by Bechtel *et al*. [19], and also in a study by Dzemali *et al*. [18], using the so-called Syncrus wires, a similar version of the TADpole wires. The authors concluded that this treatment is expected to reduce hospital length of stay and therefore hospital costs, and improve patient outcomes. These promising systems could not gain any further market penetration, as the company providing TADpole and Syncrus wires was acquired by another company that discontinued these product lines.

In our retrospective study, bi-atrial pacing was not the primary goal but was performed when atrial pacing was necessary. An overall review of the history of atrial pacing proved that bi-atrial pacing is the most successful treatment of all pacing methods to prevent postoperative atrial fibrillation [4, 5, 6, 7, 8, 9]. A review by Mitchell [10] further details the results of an evaluation of 12 trials of prophylactic atrial pacing involving 1708 patients. Overall, when combining all results regardless of atrial pacing site (right, left, or bi-atrial), or the pacing algorithm used, prophylactic atrial pacing significantly reduced the incidence of postoperative pacing. The meta-analysis from Crystal [11] demonstrated that when all pacing algorithms were combined, there was a statistically significant difference achieved with bi-atrial pacing, reducing the postoperative hospital length of stay by 1.54 days. In another meta-analysis from Burgess [12] it was shown that, while all pacing sites and algorithms combined are beneficial, the only significant result was seen in the bi-atrial pacing group, which reduced atrial fibrillation from an average of 35.3% in the control group to 17.7% in the paced group (OR 0.44, 95% CI 0.31–0.64). In view of these positive outcomes, the European Society of Cardiology (ESC) together with the European Association of Cardio-Thoracic Surgery (EACTS) has added bi-atrial pacing as a recommendation in their guidelines for postoperative treatment of patients to prevent atrial fibrillation. However, studies involving temporary bi-atrial pacing can also be difficult to conduct with the pacing wires that are currently on the market: bi-atrial pacing requires the placement not just of the standard ventricular and right-atrial pacing wire, but also requires a left-atrial pacing wire or wires. These left atrial pacing wires are difficult to attach on the left atrium.

In our study we investigated the ${\mathrm{DefiPace}}^{\text{TM}}$ system, which combines the functions of bi-atrial pacing and low-energy cardioversion in one single hand-held device. This gives the physician the option to treat the postoperative patient without time delay with low-energy cardioversion in case of the onset of postoperative atrial fibrillation, or to administer overdrive bi-atrial stimulation to prevent POAF. We investigated the initial clinical application of this new system, particularly the handling of the atrial pacing and cardio-version wires TMA® in the context of low-energy cardioversion. Placement of these atrial electrodes seems to be much easier and safer than those described in the previous studies using the TADpole and Syncrus wires. The reason might that multiple stitching of tissue is not necessary due to the zigzag shape of the distal end, and the multiple wire options with variable fixation mechanisms. In our opinion, the left atrial placement of the electrodes via the transverse sinus is a safe approach, especially during OPCAB surgery. The electrodes had a stable position, and placement was easy with acceptable sensing and threshold values. Since bi-atrial pacing is triggered by sensing of the right electrode only, sensing of the left atrial electrode is not an important parameter but documents its safe position. When using a right internal thoracic artery, which we passed through the transverse sinus as a pedicled graft to the left circumflex vessels, no electrode was placed through the transverse sinus to the left atrium. However, the anode (zigzag) must still be placed carefully. The concept of bi-atrial cardioversion is already proven with good results and can be performed without anesthesia or only a little sedation [13, 15, 18]. Liebold treated 20 patients for atrial fibrillation (AF) with a shock energy up to 10 J. In 80% of the patients, AF converted successfully to sinus rhythm with a mean shock energy of 5.2 ± 3 J. Only 6 of the 20 electrically treated patients (30%) required sedation or analgesia. At our clinic, short-acting anesthesia was administered because of the limited experience. Only 2 patients had internal low-energy cardioversion. Even with a shock energy of 3.5 J, our patients had visible muscle contractions. Therefore, we continued to administer a short sedation with propofol to subsequent patients.

Since we have only a few patients in the database who have required atrial conversion, we cannot give conclusive guidance on the optimal placement of the electrodes. However, a study is currently underway to answer this and other questions. The follow-up PMCF Registry Study (ClinicalTrials.gov Identifier: NCT04804748) will be conducted as an international multicenter study with 10*–*12 participating centers within Europe. In brief, in the control arm of the study standard of care will be documented in at least 150 consecutive patients (based on statistical analysis) that are eligible for cardiac surgery, of which at least 50 patients develop postoperative atrial fibrillation. Those patients serving as a control group will be implanted with standard Osypka TME pacing wires only, which are not intended for bi-atrial pacing or atrial defibrillation. In the second arm (treatment group), about 300 patients will be recruited. The TMA® electrodes will be implanted and connected to the ${\mathrm{DefiPace}}^{\text{TM}}$ device. Recruitment ends when at least 100 patients developed postoperative atrial fibrillation and have been treated with ${\mathrm{DefiPace}}^{\text{TM}}$ (cardioversion, bi-atrial pacing or both). Patients from both arms who developed postoperative atrial fibrillation will be followed up for 30 days. Several defined study criteria will be observed such as AF burden (time in atrial fibrillation), length of ICU stay, and others. All referenced studies focused on the therapy of PAOF. Of note, an interesting paper [21] investigated on the relationship between preoperative atrial conduction abnormalities and POAF in cardiac surgery patients without a history of AF. The study demonstrated that premature atrial S2 beats accentuated conduction abnormalities in the posterior left atrial wall of cardiac surgery patients who developed POAF. The findings might have influence on future investigations regarding understanding and therapy of PAOF.

## 5. Conclusions 

The ${\mathrm{DefiPace}}^{\text{TM}}$ system with the TMA® epicardial electrodes can be used safely and with efficacy for single or bi-atrial pacing in patients undergoing cardiac surgery. Further studies are in preparation for the efficacy of POAF treatment with this system.
